# Items of Local Interest

**Published:** 1914-08

**Authors:** 


### Items of Local Interest.

DR. BURRELL, of Lampasas, Texas, died on July 26th. Dr. Burrell was very much loved by all who knew him, and he will be sadly missed by his friends.

GOOD OPPORTUNITY for physician and surgeon in Willacy county, Texas. Salary and practice. High class recommendations required. Address "County Judge," Sarita, Texas.

DR. AND MRS. FRANK PASCHAL have returned from their summer trip, having spent part of the time in Atlantic City. The greater part of their vacation was spent in New York with their son, Frank Paschal, Jr.

ARE YOU THINKING OF BUYING ANYTHING?—If you are, look through the pages of the JOURNAL and see if you cannot find what you want. When you write out your order, take the time to add an extra line and tell them you saw their advertisement in the TEXAS MEDICAL JOURNAL.

Dr. H. I. Cook, formerly of Sweetwater, Texas, is now located at Mineral Wells.

---

Dr. W. R. Smith, of Loraine, has returned from a month's stay in Chicago, Detroit and Rochester.

---

At the thirty-eighth meeting of the Royal Commission on Venereal Diseases, evidence was given by Sir William Osler. Sir William stated that the official statistics of deaths, published by the Registrar General, were, for instance, in 1910 the deaths from syphilis in England and Wales were given as 1649, but this was a very unsatisfactory and incomplete estimate. It was necessary to take into consideration and add a very large number of deaths appearing under other descriptions. If regard were had to these, he thought it would be safe that of the killing diseases syphilis came third or fourth. It was a part of the work of the governors of hospitals to provide for these diseases, and they ought not to be left out; in the past they had been too much neglected by the charitable public. He was in favor of compulsory notification. He thought that it would have an immense effect if the public were instructed, by means of lectures, regarding these diseases. Lectures of this kind, he thought, should not be given by a layman, but by a well-trained medical man, who should be provided with proper diagrams and slides. It would be a very useful thing if the lecture could be widely given to the senior students of the big public schools, in the universities, and at large institutions employing many persons.

---

Examination Requirements.—At the last meeting of the Board of Medical Examiners the following interesting announcement was made: Beginning with the coming college year the examination board will require of students that they secure a permit from the Board, based upon an application, showing they have 14 units in a standard affiliated high school, seminary or academy and an additional college year consisting of 5 college units; one each in biology, chemistry, physics and a modern language; the other to be elective. With the additional provision that they may be conditioned on the college year or a part thereof until the beginning of their junior year in medicine. In other words, students may matriculate with 14 accepted units and sufficient credits on their college year as will enable them to complete their full college year before the beginning of the junior year.

Dr. Murchison, President of the First National Bank of Athens, Texas, died July 18, 1914. Dr. Murchison was well known in East Texas, and he was connected with a number of other banks.

---

Dr. Shadrach Burton Dies.—Dr. Shadrach Burton, aged 83 years, died at his home in Dallas, Texas, Monday evening. Dr. Burton was a retired physician, and is survived by a son, Arthur Burton, and daughter, Miss Emily Burton, both of this city. The body is being held by the Henninger-Brewer Undertaking Company, pending arrival of relatives.

---

The State Medical Association of North Carolina has gone on record as opposed to the use of alcohol. The following resolutions were unanimously adopted by the State Association at the last meeting:

"*Be it resolved,* That the Medical Society of North Carolina will use its efforts to discourage the use of alcohol in any form as a beverage.

"*Resolved,* second, That it is the sense of this Society that a member of the profession who does promiscuous or unnecessary prescribing of whisky, either to patients or non-patients, is violating one of the principles of our profession, and is deserving of censure.

"*Resolved,* third, That alcohol as a drug can be eliminated from the pharmacopeia, without in any degree crippling the efficiency of the doctor's armamentarium."

---

Falls County Doctors Meet at Chilton.—The Falls County Medical Society held a meeting July 6th at Chilton, with the following members present: Dr. Rice, J. C. and F. H. Shaw, Buie, Earle, Whitten, Shankle, Curry, J. W. and O. Torbett. The following visiting doctors were present: H. T. Aynesworth, Waco; O. F. Gober, J. J. Terrill, G. C. Kindley, Temple; J. H. Gainey, W. E. McGee, Mooreville; R. Goggin and Mercer, Chilton; Moore, Lott; W. H. Allen, B. M. Torbett, Marlin.

A very interesting program was presented. Dr. Aynesworth read an interesting and instructive paper on "The Nose and Throat as a Source of Infection." Dr. Goggin discussed "Mucous Colitis," and Dr. Gainey read an interesting paper on "Summer Diarrhea."

The doctors and citizens of Chilton served a delicious and refreshing lunch after the program was over.

ON JULY 10TH occurred the death of Robert Boyd, of Weatherford, Texas. Dr. Boyd is survived by a wife and four children.

---

DOCTORS WORK ON YOUNG DWARF.—The Blair County Medical Society, by the use of thyroid glands of lambs and sheep, is transforming a young dwarf of Hollidaysburg, Pa., Jules Schroeder, aged four years, into natural proportions. The boy is suffering from cretinism, but under treatment his arms and legs are steadily lengthening, it was said by members of the society last night. The experiment is being watched with great interest by the medical fraternity.

---

ABRIDGING THE DOCTOR'S ORDERS.—A bricklayer lay ill, and the doctor having done what he could told the man's wife to take his temperature in the morning. Calling the next day, the doctor asked if his instructions had been followed.

"Well, he hadn't a 'tremometer' in the house," the good woman replied, "but I put a barometer on his chest and it went up to 'very dry.' So I gave him a bottle of beer and he's gone to work."—*Chicago Herald.*

---

RICHMOND, VA., July 9, 1914.

*Mrs. F. E. Daniel, Austin, Texas.*

MY DEAR MRS. DANIEL: It is with deep sorrow that I have just learned of the death of dear Dr. Daniel. Please allow me to tender you my sincere sympathy in your great bereavement, and to say that the profession and the editorial fraternity have lost one of the brightest, bravest and best members that ever graced their ranks. He was not afraid to do his duty in the face of the whole world, and it takes a *great* man to do this. I am glad to know that you have resolved to keep his memory green by continuing the great work he had so successfully inaugurated and so long conducted with high honor to himself and unmeasured benefit to the profession and to humanity. I know you will succeed, and I feel proud of your announcement that you are not asking for support of the TEXAS MEDICAL JOURNAL on account of sympathy but for its merits. His mantle has descended upon one worthy of its glorious associations, and I wish you heaven's blessings in all of your efforts.

Sincerely your friend,

C. A. BRYCE.

Editor of the *Southern Clinic.*

Dr. Delano R. Aves is now permanently located at El Paso, Texas.

---

Dr. T. A. Morrison has recently moved from Santa Anna, and is now doing business at Grosvenor, Texas.

---

Dr. E. S. Smith, of Crosbyton, is adding some very valuable improvements to his home, in the form of screened porches, cement sidewalks, and a windmill, which supplies water to every part of the house and affords fire protection. When completed, the doctor will have one of the most attractive and up-to-date homes in Crosby county.

---

J. B. Nitschke, State Inspector of Masonry, left Austin June 29th for San Antonio to make preparations for starting work for the construction of two new ward buildings and a laundry on the grounds of the Southwestern Insane Asylum. The contract for these structures was let Saturday and will cost $200,000. The two new wards will afford accommodations to 550 patients.

---

A Significant Comment.—"Whoever," remarks the *McGregor Mirror,* and it is echoed by the *Abilene Daily Reporter*—"Whoever is elected Governor of Texas, we trust he will have foresight and judgment enough to perceive the glaring truth that Texas needs Dr. M. M. Carrick as State Health Officer." Dr. Carrick has done a great work in Texas, seeking no public recognition of the fact. He has been the power behind the throne in the clean-city campaign, which has resulted in untold good to every part of the State. The next Governor of Texas would find practically general approbation of his act did he name Dr. Carrick to the high and important place.—*State Topics.*

This suggestion will meet with the approval of the many people who have been connected with the clean-up movement in Texas. If the next Governor of Texas wants to strike a popular chord, let him take up this suggestion and secure the services of Dr. Carrick, that he may continue to clean up Texas as one having authority of the State to back up his efforts. Let us get Texas cleaned up before there is a chance for the bubonic plague to invade our territory.—*Temple Telegram,* July 24, 1914.

---

Dr. L. P. Allison and family, of Brownwood, have just returned from a five weeks' visit in the North. While away the doctor visited the great hospitals of Chicago, New York and other cities and attended the National Medical Association at Atlantic City.

---

Contract for the improvement of the Texas Tuberculosis Sanitarium at Carlsbad was awarded recently to a Fort Worth firm. A concrete power house, store house, library and auditorium are to be built at a cost of $22,600.

---

An effort is being made to build a sanitarium at Ladonia. With seven skilled physicians, an abundant supply of valuable mineral water, and many other natural advantages, we see no reason why this venture should not prove a complete success.

---

Dr. M. M. Carrick, of Dallas, the noted sanitation expert, has recently been called to Temple for a conference with the City Clean-up Commission, which was recently appointed by the city council to take charge of and direct a general clean up of the city. The Clean-up Commission has shown fine business ability, and we congratulate the people of Temple on having such a wide-awake commission and on being able to secure Dr. Carrick's services. Maybe they want the cleanest city prize.

---

The danger from "unwrapped bread" was clearly set forth by Dr. C. C. Grein, City Health Officer of Houston, in a forceful speech before the Rotary Club of that city. The doctor stated that in New Orleans, where *all* bread *must* be wrapped, they give 17 ounces for 5 cents, which is more than is given in any other city. We quote the following from his speech, which to us is unanswerable and must appeal to every thinking person:

"If you will stop and think, you will realize that bread, above all things, should be most carefully protected, and an illustration just here will explain my position thoroughly. For instance, if you buy fruit, you usually wash it or cook it before eating. If you buy meat, you both wash and cook it; but bread—do you either wash or cook it before eating? No. It is absolutely necessary that you eat your bread just as it is received from the hands of the wagon driver, and contaminated with dust which has been collected during its delivery. It is absurd to expect the driver to keep his hands clean, but the workman in the shop can have a place to wash his hands before he wraps the bread, and in this way we protect the bread from the two most dangerous sources of contamination, viz., the hands of the driver and the dust of our streets."

Dr. Ferris A. Bass, of Brownwood, has received the appointment of Dental Surgeon to the Southern Pacific Hospital at Houston. This appointment is a distinct honor, since the Southern Pacific has the largest hospital of the kind between San Francisco and New Orleans.

---

The Paluxy Company, of which Dr. H. L. Wilder, of Glen Rose, is president, is planning great things for the people of Somervell county. The company owns 200 acres of land on Paluxy river. On this land there are three large wells, each flowing an average of sixty gallon of water per minute. A dam will be placed across one of the streams running through the place, and on this lake will be built an up-to-date sanitarium. Camping places will also be arranged. With a big new sanitarium and camping, fishing, boating and bathing to add to the attractions, we venture a guess that Glen Rose will soon be a leading health resort.

---

A wedding which is of more than passing interest in medical circles was that of Dr. Louis Daily, of Houston, and Dr. Ray Karschmer, of Denison, which occurred June 9th at the home of the bride's parents. Dr. Karschmer graduated from the Medical Department of Galveston in 1913, and was awarded first honors in the State. President Mezes of the University in his address to the graduating class finished his eulogy to the splendid achievement of the Denison girl by saying, "She has honored the University of Texas." Not content with having graduated, Dr. Karschmer spent last year in various hospitals in the East, taking special work in eye, ear, nose and throat treatment, and successfully passed examinations in the polyclinic of Philadelphia, being the first and only woman doctor to be awarded a diploma of proficiency.

Dr. Daily is from Wishneva, Russia. He graduated from the University of Texas Medical Department in 1910. He spent the following year in practice in a California hospital, then returned to Houston and established his practice.

To these "two souls with but a single thought" we wish to extend our heartiest congratulations, and wish for them all the realization of all their rosy-hued dreams.

---

Central Texas District Medical Society Meets in Hillsboro.—With about fifty of the membership present, the Central Texas District Medical Society was called to order by the President, Dr. F. D. Sims, of Abbott, Texas. The general program was arranged in sections as follows: General Medicine—Dr. Joe E. Dildy, Lampasas, Chairman; Dr. Ben F. Smith, Hillsboro, Secretary. Surgery—Dr. W. D. Fountain, Corsicana, Chairman; Dr. W. M. Sherwood, Temple, Secretary. Gynecology and Obstetrics—Dr. R. J. Alexander, Waco, Chairman; Dr. Frank Shaw, Marlin, Secretary.

The following papers were read and discussed the first day of the meeting:

General Medicine—S. P. Rice, Marlin, President; H. F. Connally, Waco, Secretary; Dr. Joe E. Dildy, Hillsboro, Chairman; Dr. Ben F. Smith, Hillsboro, Secretary. "Early Diagnosis, Treatment and Control of Pellagrins," Dr. W. B. Cross, Corsicana. "Ophthalmia Neonatorum," Dr. W. W. Fowler, Hamilton. "Some Harmful Effects of Intestinal Toxins," Dr. J. J. Terrell, Temple. "The Diagnosis of Pellagra without the Eruption," Dr. Wilmer Allison, Fort Worth. "Tetanus, with Report of Case Treated with Serum." Dr. C. W. Davis, Waco. "The Pathological Physiology of Tuberculosis and Its Relation to Tuberculin Therapy," I. S. Kahn, San Antonio. "The Value of Sphygmomanometer to the General Practitioner," H. H. Lanham, Waco.

During the evening session the following subjects were discussed: "Ophthalmia Neonatorum," Dr. W. Fowler, Hamilton. "Mucous Colitis," Dr. Roy Goggans, Chilton. "The Surgical Treatment of Trachoma," Dr. W. R. Thompson, Fort Worth.

After which refreshments were served and the meeting declared adjourned until the following day.

Those in attendance at the opening session were as follows: Dr. H. F. Connally, Waco; Dr. J. E. Dildy, Lampasas; Dr. Ben F. Smith, Hillsboro; Dr. H. N. Lanham, Waco; Dr. J. E. Boyd, Hillsboro; Dr. J. J. Terrell, Temple; Dr. J. F. Holland, Itasca; Dr. C. H. Harris, Fort Worth; Dr. J. M. Frazier, Belton; Dr. W. W. Lowrey, Hillsboro; Dr. T. D. Sims, Abbott; Dr. I. W. Jenkins, Penelope; Dr. J. M. Burgess, Waco; Dr. C. C. Davis, Hillsboro; Dr. J. B. Shelmire, Dallas; Dr. E. D. Ward, Blum; Dr. J. A. Speed, Itasca; Dr. D. K. Robinson, Itasca; Dr. W. D. Fontaine, Corsicana; Dr. W. D. Cross, Corsicana; Dr. F. M. Douglas, Itasca; Dr. J. S. McCawan, Oceola; Dr. J. L. Montgomery, Aquilla; Dr. A. M. Center, Waco; Dr. H. L. Ivy, Whitney; Dr. H. L. Wilder, Whitney; Dr. Edwin Vaughan, Hillsboro; Dr. C. M. Davis, Waco; Dr. H. C. Brock, Waco; Dr. Roy Loggan, Chilton; Dr. J. W. Matlock, Frost; Dr. D. T. Eastland, Waco; Dr. M. W. Brian, Hillsboro; Dr. I. S. Kahn, San Antonio; Dr.

G. D. Bond, Fort Worth; Dr. J. C. Stevens, Richland; Dr. T. H. Shaw, Marlin; Dr. H. S. Mahaffy, Hillsboro.

---

Dr. and Mrs. W. B. Adams, of Electra, have gone to Tennessee, where the doctor will take a post-graduate course at Vanderbilt.

---

Dr. J. H. Allred, of Winters, Texas, after a long and severe illness, is able to be out again, much to the delight of a host of friends and patrons.

---

Doctors Meet at Kerrville.—The Kerr-Kendall-Gillespie-Bandera County Medical Society met in Kerrville on July 8th. The morning was spent in clinical and operative work at the hospital by Dr. Scott and Dr. Palmer. During the afternoon a scientific program was presented at Pampell's Hall.

---

The De Witt County Medical Society met in Yorktown Wednesday, July 19th. The following physicians were present and participated: Drs. Lackey, Muegge, Froboese and Duckworth, of Cuero; Mernitz, of Nordheim and Westphal; Nowiesskie and Eckhardt, of Yorktown. The society will meet next month in Cuero. During their stay the visiting physicians were given a nice banquet.

---

Twenty of the leading physicians of Waco declare that the typhoid fever scare is groundless. They say the scare has grown out of the fact that there is a great deal of catarrhal fever, and that in the first stages of this fever it is sometimes hard to diagnose, and the symptoms are very similar to those of typhoid fever. However, the blood test, which is absolutely authentic, has proven the absence of typhoid.

---

Dr. and Mrs. J. H. Mitchell, of Dallas, recently celebrated their golden wedding. Dr. Mitchell is one of the oldest physicians in the State, having located in Rockwall in 1862, when he practiced medicine over a radius of sixty miles. During the Civil War, Dr. Mitchell served as assistant surgeon under General Price. Mrs. Mitchell tells of a time when there was not a window glass in any of the houses, and when she would ride horseback twenty or more miles to a dance. They were surrounded on this happy occasion by their children and grandchildren.

Dr. I. E. Clark, of Schulenburg, has been re-elected to the Texas Senate.

Dr. I. L. Maxwell, of Wylie, is soon to erect a handsome residence, which is to be his permanent home.

Dr. H. C. Homan, of McGregor, recently had the misfortune to break both bones in the right forearm, while cranking his automobile.

Dr. I. A. Langston, of Waco, Texas, has returned from a five weeks' visit in Chicago. While away the doctor spent the time in sightseeing, and attending medical lectures and clinics.

After a conference with county and local health officers, Dr. J. H. Thorne, field director of the Hookworm Commission, decided to postpone the hookworm campaign in Red River county until fall. At present, Bell county is the seat of activity of Dr. Thorn and his assistant, Dr. J. C. Adams.

Dr. H. L. Middlebrook, of Alpine, has let the contract for a very handsome residence.

---

Dr. T. H. Harrell, of Martindale, writing to the Journal says, among other things: "I thought all *good doctors* were long since subscribers to the 'Red Back.' It was the first journal I put on my list when I began the practice of medicine. You see by that I began right. So long as you are connected in any way with this, the best of magazines, I shall read it."

---

The trustees of the American Medicine gold medal award respectfully announce that the medal for 1914 has been conferred upon Dr. George W. Crile, of Cleveland, Ohio, as the American physician who in their judgment has performed the most conspicuous and noteworthy service in the domain of medicine and surgery during the past year.

---

The September issue of the "Red Back" will contain an article on "Drug Taking Doctors," by Dr. T. D. Crothers. Dr. F. C. Floeckinger will have an illustrated article on "Important Factors to be Considered in the Diagnosis and Treatment of Carcinomata of the Breast." An article on "Auto-intoxication," by Dr. James McLaughlin, who has just returned from the New York Post-Graduate School of Medicine, will be another interesting feature of the September issue.

The Department of Eugenics will contain the following interesting papers: Poisoned Darts; The Injury of the Obsession of Sex on the Mind; Too Late; Suggestions for Marital Hygiene; Is Sex the Strongest Instinct? Background for Mental Development.

---

Doctor.—If you have anything in physicians supplies that you do not use or need, why not advertise it and sell it to some one who does need it? It may be there is some physician who is in need of the instrument, book or physician's case that you need no longer. He would be glad to pay you a reasonable price for the article, and you would have helped him as well as yourself. Or if you wish to buy anything and save money on the purchase you can doubtless get it from some other physician by letting your wants be known through these columns. Physicians desiring new locations or partnership practice, or localities needing a physician, will find the Texas Medical Journal a valuable advertising medium.

COTTAGE FOR CONSUMPTIVES.—A cottage for consumptives has been erected at the North Texas Hospital for the Insane for the special use of consumptive patients of that institution, which will be segregated from the other patients as much as possible hereafter.

---

WANTED AND FOR SALE.

(Rates: Three cents per word in advance.)

WANTED.—Good location by young physician. Address "Dr. A.," care of "Red Back."

---

WANTED—To buy a complete stock of drugs and sundries with established trade. Address "Dr. H. B. S.," care of TEXAS MEDICAL JOURNAL.

---

WANTED—A mature woman to take entire charge of a household, small family of grown people. Must be strong, good natured, willing, honest and practical. A good home and good wages to the right party. Address "Dr. K., care of TEXAS MEDICAL JOURNAL.

Dr. E. B. Brannin, of Dallas, has just returned from an Eastern trip. While away, he attended medical clinics in New York, and the meeting of the A. M. A. in Atlantic City. He has just completed a post-graduate course in Johns Hopkins University.

